# Molecular and Pharmacological Evidence for the Expression of Multiple Functional P2 Purinergic Receptors in Human Adipocytes

**DOI:** 10.3390/molecules27061913

**Published:** 2022-03-16

**Authors:** Marco Rossato, Francesca Favaretto, Marnie Granzotto, Marika Crescenzi, Alessandra Boscaro, Angelo Di Vincenzo, Federico Capone, Edoardo Dalla Nora, Eva Zabeo, Roberto Vettor

**Affiliations:** 1 Department of Medicine-DIMED, Clinica Medica 3, School of Medicine, University Hospital of Padova, 35128 Padova, Italy; francesca.favaretto@unipd.it (F.F.); marnie.granzotto@unipd.it (M.G.); marikacrescenzi@hotmail.com (M.C.); aboscaro@casacura.it (A.B.); divincenzoang@gmail.com (A.D.V.); caponefederico@hotmail.com (F.C.); eva.zabeo@aopd.veneto.it (E.Z.); roberto.vettor@unipd.it (R.V.); 2 Medical Department, University Hospital of Ferrara Arcispedale Sant’Anna, 44121 Ferrara, Italy; dlldrd@unife.it

**Keywords:** ATP, P2 receptors, adipocyte, adipose tissue, inflammation, IL-6

## Abstract

Extracellular ATP exerts important functions as an extracellular signaling molecule via the activation of specific P2 purinergic receptors (P2X and P2Y). We investigated the expression of the different P2 receptors and their possible functional activation in human adipocytes in primary culture. We performed molecular expression analysis of the P2 receptors in human mature adipocytes; examined their functional activation by different nucleotides evaluating [Ca^2+^]_i_ modifications and IL-6 secretion, and determined the ability of adipocytes to release ATP in the extracellular medium. Human adipocytes express different P2X and P2Y receptors. Extracellular ATP elicited a rise in [Ca^2+^]_i_ via the activation of P2X and P2Y receptor subtypes. Human adipocytes spontaneously released ATP in the extracellular medium and secreted IL-6 both at rest and after stimulation with ATP. This stimulatory effect of ATP on IL-6 secretion was inhibited by pre-incubation with apyrase, an ATP metabolizing enzyme. These results demonstrate that human adipocytes express different P2X and P2Y receptors that are functionally activated by extracellular nucleotides. Furthermore, human adipocytes spontaneously release ATP, which can act in an autocrine/paracrine fashion on adipocytes, possibly participating in the regulation of inflammatory cytokine release. Thus, P2 purinergic receptors could be a potential therapeutic target to contrast the inflammatory and metabolic complications characterizing obesity.

## 1. Introduction

Adenosine 5′-triphosphate (ATP) is known as the primordial molecule providing the energy for all living cells [1]. Besides this fundamental role, ATP has gained importance as an extracellular signaling molecule and there is now no doubt that ATP exerts widespread and specific actions in the regulation of a variety of cellular functions in many different tissues [2,3]. Since the first proposal by Geoffrey Burnstock of the possibility that ATP could act as an extracellular signaling molecule activating specific plasma membrane receptors on the target cells [2,3], the studies regarding the actions of purines and pyrimidines have expanded so widely that there is no cell type where the “purinergic system” has not yet been studied [3].

The effects of extracellular ATP on target cells are mediated via the activation of specific plasma membrane receptors named P2 purinergic receptors, which, on the basis of pharmacological, functional, and molecular data, have been grouped into two families named P2X and P2Y [4]. Presently, the P2X family is composed of seven cloned subtypes (P2X1 to P2X7) while the P2Y family is composed of eight cloned subtypes [4]. While P2X the receptors are plasma membrane channels directly opened by extracellular ATP and permeable to monovalent and divalent cations, the P2Y receptors are seven membrane-spanning G-protein coupled receptors, the activation of which triggers the generation of inositol 1,4,5-trisphosphate and the release of calcium (Ca^2+^) from internal stores [4,5].

Previous pharmacological studies have described different effects of extracellular ATP in adipocytes, such as the inhibition of leptin secretion, the inhibition of glucose transport, the induction of aromatase activity, the stimulation and inhibition of lipogenesis, and the modulation of ion channel activity [6,7,8,9,10,11,12,13,14]. All the data indicate that extracellular nucleotides and P2 receptors modulate important functions of adipocytes. However, to date, only a limited number of pharmacological studies have tried to characterize the different P2 receptors expressed in adipocytes (mainly in murine brown adipose tissue) [6,7,8,9,10,11,12] and no clear information is available on the actual molecular identity of the different P2 receptors expressed in human white adipose tissue. The various P2 receptors have many functional properties, but these may overlap, and since many cells express multiple P2 receptor subtypes, a precise pharmacological identification of the native P2 receptors expressed in adipose tissue is difficult since ATP, for example, is the primary endogenous ligand of all P2X receptors [3].

In the present study, we performed molecular characterization of the P2X and P2Y receptor expression in human subcutaneous and visceral adipose tissue, together with an evaluation of the functional effects of ATP in human adipocytes in culture.

## 2. Results

### 2.1. Human Adipocytes/Adipose Tissue Express mRNA for Multiple P2X and P2Y Receptor Subtypes

**Figure 1:**
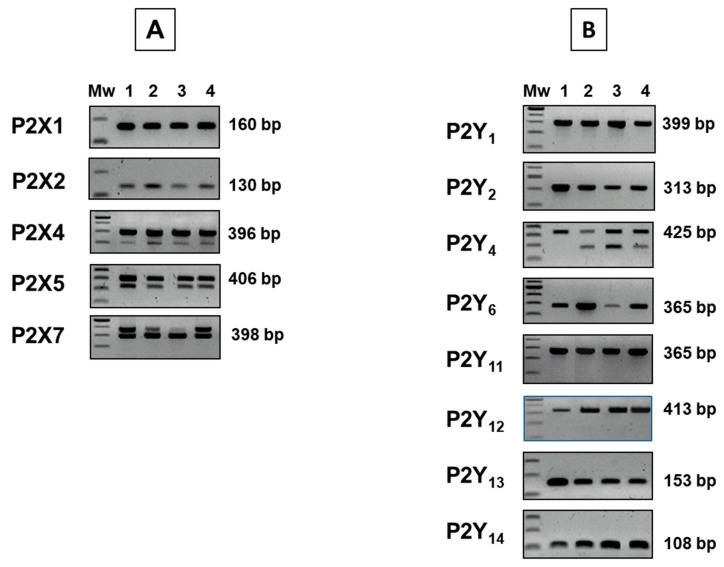
P2X (**A**) and P2Y (**B**) purinergic receptor subtype mRNA expression in human adipose tissue. Mw: marker weight; lane 1: positive control; lane 2: in vitro differentiated mature adipocytes; lane 3: subcutaneous adipose tissue; lane 4: visceral adipose tissue.

We first investigated the expression of P2 receptor subtype genes in ex vivo human subcutaneous and visceral adipose tissue and in in vitro differentiated human adipocytes, and found that adipose tissue expresses many of the known P2X and P2Y receptor subtypes (Figure 1). P2X3 and P2X6 were not detected in either ex vivo adipose tissue samples or in vitro differentiated human adipocytes. The use of a specific primer set for P2X4, P2X5, P2X7, and P2Y_4_ highlighted the presence of multiple amplicons in addition to those of the expected size (Figure 1). In particular, those from P2Y_4_ were found only in adipose tissue and mature adipocytes (Figure 1). All fragments generated by PCR have been sequenced, confirming the reaction specificity. Using ClustalW software (http://www.ebi.ac.uk/Tools/msa/clustalw2/) and genomic databases (http://www.ncbi.nlm.nih.gov/nuccore and http://www.ensembl.org/index.html, accessed on 21 September 2021), we identified that the shorter fragment of P2X4 resulted from the deletion of exon 5 (97 bp). The shorter amplicon derived from P2X5 PCR was specific and lacking a fragment of about 84 bp, part of exon 8 (134 bp). For P2X7, we identified that the PCR reaction identified several splice variants of P2X7 gene, a longer band matching with accession numbers AY847299, AY847303, AY847298, and AY847301.

### 2.2. ATP and UTP Induce [Ca^2+^]_i_ Changes in Human Adipocytes

**Figure 2:**
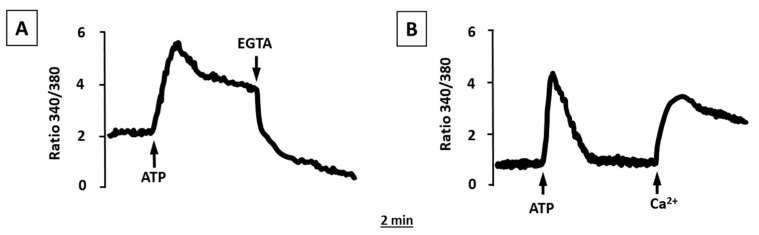
Effects of ATP on [Ca^2+^]_i_ concentrations in human differentiated white adipocytes. Human adipocytes, grown and differentiated in adipogenic medium on glass coverslips, were loaded with fura-2/AM, as described in the Section 4. (**A**) Adipocytes suspended in Ca^2+^-containing medium. (**B**) Adipocytes suspended in Ca^2+^-free medium. Where indicated, ATP (100 μM), EGTA (1 mM), and Ca^2+^ (1 mM) were added. Traces are representative of the fluorescence ratio F340/F380 of a typical experiment from three.

As anticipated by the expression of both P2XRs and P2YRs, and as previously reported [15], the stimulation of human adipocytes with ATP caused an increase in [Ca^2+^]_i_ (Figure 2A) characterized by an early rapid peak, presumably due to [Ca^2+^]_i_ release from intracellular stores, and a sustained plateau, likely due to an influx of Ca^2+^ from the extracellular medium through the plasma membrane. Indeed, the addition to the extracellular medium of EGTA, a Ca^2+^-chelating agent, rapidly abolished this sustained plateau (Figure 2A). Incubation in a Ca^2+^-free medium had no significant effect on the early [Ca^2+^]_i_ peak, but fully abrogated the sustained plateau that was rapidly activated after Ca^2+^ addition in the external medium (Figure 2B).

**Figure 3:**
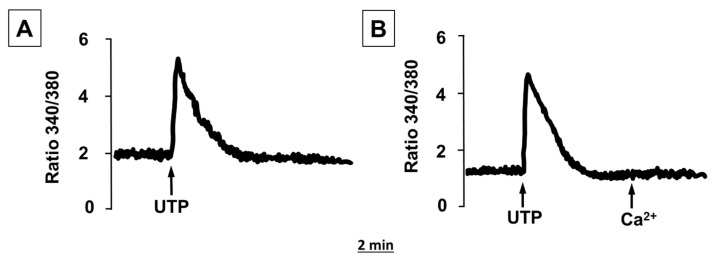
Effects of UTP on [Ca^2+^]_i_ concentrations in human differentiated white adipocytes. Human adipocytes, grown and differentiated in adipogenic medium on glass coverslips, were loaded with fura-2/AM as described in the Section 4. (**A**) Adipocytes suspended in Ca^2+^-containing medium. (**B**) Adipocytes suspended in Ca^2+^-free medium. Where indicated, UTP (100 μM) and Ca^2+^ (1 mM) was added. Traces are representative of the fluorescence ratio F340/F380 of a typical experiment of three.

Human adipocytes were also sensitive to stimulation with UTP (Figure 3A); although, unlike ATP, adipocyte stimulation with UTP evoked a rise in [Ca^2+^]_i_ coming from internal stores since in the absence of extracellular Ca^2+^, the effects of UTP were unchanged (Figure 3B).

**Figure 4:**
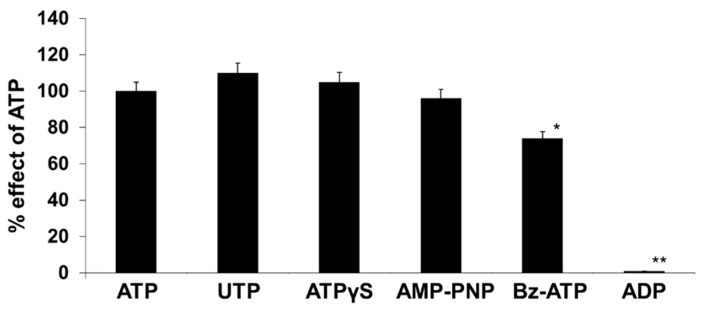
Effects of different nucleotides (100 µM) on intracellular calcium concentrations in human adipocytes. The effects of the different nucleotides have been normalized to those obtained with ATP. * *p* < 0.05; ** *p* < 0.0001.

Among the different purine and pyrimidine nucleotides, ATP, ATPγS, UTP, and α,β-methylene ATP induced a rise in [Ca^2+^]_i_ in human adipocytes, suggesting the functional activity of both P2XR and P2YR (Figure 4). ATPγS, a non-hydrolyzable ATP analog, induced a rise in [Ca^2+^]_i_ quite similar to that induced by ATP, thus suggesting that ATP itself, rather than its degradation products (ADP, AMP, adenosine), is the native P2 receptor agonist in human adipocytes. Similarly, the addition of ADP had no effect on inducing the [Ca^2+^]_i_ increase in human adipocytes (Figure 4).

**Figure 5:**
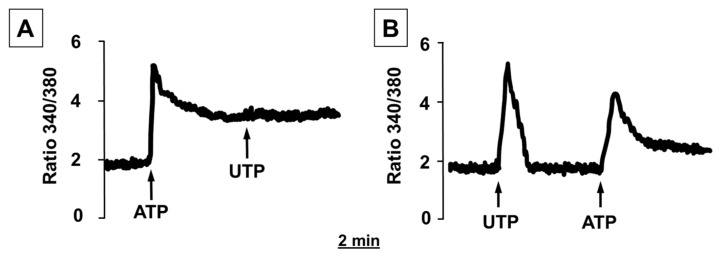
(**A**) Stimulation with ATP inhibits the effects of UTP on [Ca^2+^]_i_ concentrations in human differentiated white adipocytes. (**B**) Stimulation with UTP does not inhibit the effects of ATP on [Ca^2+^]_i_ concentrations in human differentiated white adipocytes. Human adipocytes, grown and differentiated in adipogenic medium on glass coverslips, were loaded with fura-2/AM, as described in the Section 4. Where indicated, ATP (100 μM) and UTP (100 μM) were added. Traces are representative of the fluorescence ratio F340/F380 of a typical experiment from three.

In order to characterize the contribution of the different P2 receptor subtypes to the [Ca^2+^]_i_ increase in human adipocytes, we performed cross-desensitization experiments. Figure 5A shows that the stimulation of human adipocytes with ATP inhibited the increase in [Ca^2+^]_i_ induced by UTP. When the reversal experiment was performed, only a reduction in the peak of the ATP-stimulated signal was observed (Figure 5B). 

The observations that ATP inhibits UTP effects while UTP does not interfere with the effects of ATP suggest that the ATP-induced Ca^2+^ rise was due to the activation of both P2X and P2Y receptor subtypes, while UTP was active only on the P2Y receptors, probably the P2Y_2_ and P2Y_4_ subtypes that are expressed in human adipocytes and that have been previously reported to be activated only by UTP [15]. 

### 2.3. Extracellular ATP Induces IL-6 Release from Human Adipocytes

**Figure 6:**
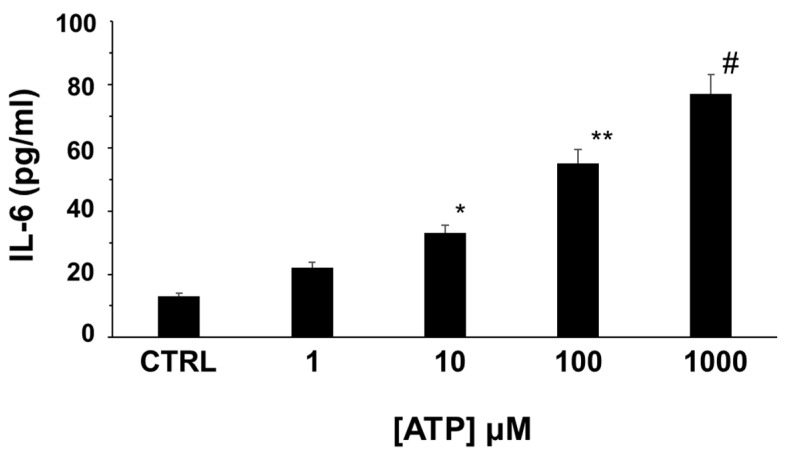
Effects of the stimulation of human white adipocyte with different concentrations of ATP on IL-6 secretion. * *p* < 0.05; ** *p* < 0.01; # *p* < 0.001.

We explored the role of extracellular ATP in modulating IL-6 release in in vitro differentiated human adipocytes. As shown in Figure 6, IL-6 is detectable in the external medium of isolated cultured human adipocytes at basal conditions and the stimulation of human adipocytes with ATP induced the secretion of IL-6 in a dose-dependent manner (Figure 6).

### 2.4. Hydrolysis of Extracellular ATP Inhibits IL-6 Secretion by Human Adipocytes

As suggested by the detectable IL-6 concentrations at resting, unstimulated conditions, we hypothesized that, in the absence of exogenously added ATP, this nucleotide might be spontaneously released from human cultured adipocytes.

**Figure 7:**
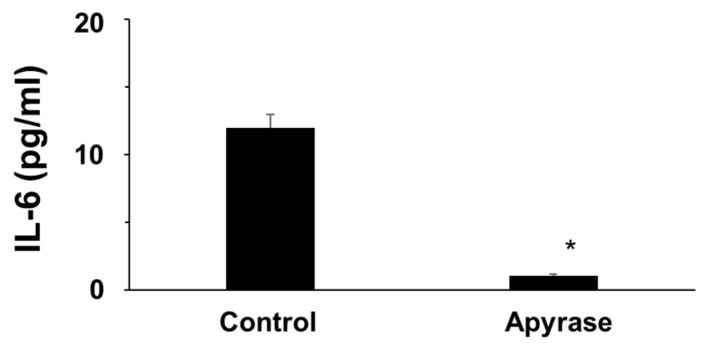
Effects of extracellular ATP hydrolysis with apyrase on IL-6 secretion by human adipocytes (* *p* < 0.001).

In this regard, the accumulation of ATP in the external medium depends on cell release and on its hydrolysis by ecto-ATPases. In agreement with the hypothesis that ATP is spontaneously released from adipocytes and thus stimulating IL-6 secretion in an autocrine/paracrine fashion, when extracellular ATP was reduced by pre-incubating adipocytes with apyrase, an enzyme that catalyzes the hydrolysis of ATP [15], basal IL-6 release was significantly blunted, confirming that human adipocytes probably release ATP in the extracellular medium at resting conditions (Figure 7). 

## 3. Discussion

Since the existence of the purinergic system was first postulated in the early 1970s, the role of ATP as an extracellular signaling molecule has been increasingly appreciated in a many different types of cells [2,3]. The presence of functional purinergic receptors in human and animal adipocytes has been previously reported, indicating that extracellular nucleotides influence the activity of these cells. In particular, it has been shown that extracellular ATP modulates leptin secretion, glucose transport, enzyme activity, lipogenesis, and ion channel activity in adipocytes [6,7,8,9,10,11,12,13,14]. Extracellular ATP exerts its effects through the activation of specific plasma membrane receptors that have been grouped into two families, namely P2X ionotropic ligand-gated ion channel receptor and P2Y metabotropic G-protein coupled receptors [3,4]. Although a few pharmacological studies have established the presence of different P2Rs in adipocytes, no detailed information on the specific P2 receptor subtypes expressed by human adipocytes is yet available.

In the present study using RT-PCR, we detected the presence of different P2X and P2Y receptor subtypes in subcutaneous and visceral adipose tissue ex vivo, and in vitro in differentiated human adipocytes. Once the expression of the different P2X and P2Y receptor subtypes in human adipocytes was determined, we proceeded to the functional analysis of these receptors, showing that they are also functionally active, since the stimulation of adipocytes with ATP elicited an increase in [Ca^2+^]_i_, characterized by a rapid initial peak and long-lasting plateau. In Ca^2+^-free medium, ATP still stimulated an increase in [Ca^2+^]_i_, but only the first rapid peak was still observed without the plateau phase, suggesting the release of Ca^2+^ from the internal stores. These observations suggest the activation of P2Y receptors since they are coupled to phospholipase C activation and Ca^2+^ release from intracellular stores. In addition, UTP induced a rise in [Ca^2+^]_i_, but this was due only to Ca^2+^ release from intracellular stores since it was quite similar both in Ca^2+^-containing and Ca^2+^-free medium, further confirming the presence of functional P2Y receptors in human adipocytes, as P2X receptors are not responsive to UTP [16]. Purinergic receptors of the P2X family are ion channel-coupled receptors that mediate ion fluxes through the plasma membrane and are responsible for the sustained Ca^2+^ influx observed in the presence of Ca^2+^ in the external medium and completely absent in Ca^2+^-free conditions [17]. The ability of BzATP, a well-known agonist of the P2X receptor with no activity on P2Y receptor subtypes [18], to induce a rise in [Ca^2+^]_i_ confirms that human adipocytes express also functional P2X receptors, the activation of which participates in the [Ca^2+^]_i_ rise induced by ATP.

Experiments involving the effects of P2 receptor desensitization showed that when adipocytes were stimulated with ATP, the response to the subsequent addition of UTP was completely absent, while when UTP was added prior to ATP, the rise in [Ca^2+^]_i_ was only reduced. These results suggest that ATP is active both in P2Y and P2X receptor subtypes, while UTP is active only at P2Y receptors. The absence of any desensitization induced by BzATP on the UTP-induced increase in [Ca^2+^]_i_ confirms that ATP and UTP act on different receptor subtypes [15].

The pathophysiological role of the functional expression of P2XR and P2YR in human adipose tissue is still elusive. In the extracellular space, ATP has been recognized as a DAMP participating in the inflammatory process leading to inflammatory cytokine production [5]. Human obesity, characterized by the expansion of adipose tissue, is considered a condition of a low-grade chronic inflammatory state characterized by the elevation of well-known inflammatory cytokines, such as TNFα and IL-6 within plasma [19]. Adipose tissue produces a wide range of cytokines that have been implicated in the regulation of metabolism, such as IL-6, a well-known pro-inflammatory mediator that has been implicated in the maintenance of the low chronic inflammatory state and participating in the pathogenesis of metabolic and cardiovascular complications afflicting obese subjects [19,20,21,22]. In this respect, IL-6 is a multifunctional inflammatory cytokine produced by many different cell types, including adipocytes, and it is well known that circulating and adipose tissue levels of IL-6 are elevated in obese subjects and linked to the metabolic and vascular complications of obesity [22,23,24]. The regulation of IL-6 secretion in adipocytes is not well known, but in many different cell types, IL-6 secretion is stimulated by extracellular ATP through the activation of P2 purinergic receptors [25]. Here, we show that extracellular ATP stimulates the secretion of IL-6 in a dose-dependent manner in human adipocytes in vitro, thus demonstrating that the purinergic system modulates the secretion of IL-6 in human adipocytes as previously shown in other cell types [25].

After the demonstration that extracellular ATP stimulates IL-6 secretion in human adipocytes, the source of extracellular ATP has to be established. We hypothesized that human adipocytes might spontaneously release ATP in the extracellular medium at resting conditions that could stimulate IL-6 secretion via P2 purinergic receptor activation on adipocytes in an autocrine/paracrine fashion. The inhibition of IL-6 secretion by human adipocytes when extracellular ATP was hydrolyzed with apyrase, a well-known ATP scavenger [15], confirmed our hypothesis. Thus, these observations make it possible to hypothesize that basal IL-6 secretion from human adipocytes in vitro observed in the present study could be due to the basal tonic release of ATP by adipocytes themselves. 

Obesity and overweight, together with their associated metabolic and cardiovascular complications, affect over 50% of the adult population worldwide [26,27,28]. It is well known that these conditions are associated with a low-grade chronic inflammatory state with abnormal cytokine secretion and the activation of specific signaling pathways, which could link obesity to metabolic and cardiovascular diseases [26,27,28]. A novel and interesting feature of the inflammatory response present in obesity is that it seems to start and reside within the adipose tissue [29,30,31,32] and it has been previously demonstrated that adipocytes can produce a number of different inflammatory cytokines [19]; since its first classification as a mediator of cell-to-cell communications, ATP and its purinergic receptors have been shown to be involved in the complex series of events initiating inflammation [33,34].

The present results might open new pathophysiological perspectives on the modulation of adipocyte activity by targeting the adipocyte P2 purinergic system in adipose tissue to tackle the inflammatory activity of adipocytes mainly in the presence of adipose tissue expansion, such as in obesity.

## 4. Materials and Methods

### 4.1. Ex Vivo Adipose Tissue Sampling and In Vitro Differentiation of Human Pre-Adipocytes to Mature Adipocytes

Biopsies of omental adipose tissue (approximately 300 mg) were collected from patients undergoing elective abdominal surgery for non-malignant abdominal disease (7 lean patients, M/F = 3/4, age 46 ± 5 yrs, BMI = 23.9 ± 1.1 kg/m^2^), which included pancreatic pseudocysts, cholecystectomy for gallbladder stones, abdominoplasty. Subcutaneous adipose tissue samples for pre-adipocyte isolation and in vitro maturation to mature adipocytes were obtained from patients undergoing elective abdominoplasty after huge weight loss due to bariatric surgery. All patients were free from malignant disease, major renal or hepatic dysfunction, diabetes, or endocrine-metabolic disorders. After collection, adipose tissue samples were washed in ice-cold saline, frozen in liquid nitrogen, and stored at −80 °C or used for stromal vascular fraction and mature adipocyte collection. For the isolation of human pre-adipocytes, subcutaneous adipose tissue samples were minced and digested for 1 h at 37 °C in a collagenase type II solution (1 mg/mL) (Sigma-Aldrich, MO, USA). The cell suspension was then centrifuged (350× *g* for 8 min), and the pellet containing stromal cells was resuspended in erythrocyte-lysing buffer, washed, and seeded in DMEM/F12 supplemented with 10% fetal bovine serum (0.7 × 10^6^ cells per well in 24-well plates). After 16–20 h for cell attachment, cultures were re-fed with a serum-free adipogenic medium containing DMEM/F12 supplemented with 33 µmol/L biotin, 17 µmol/L pantothenate, 10 µg/mL human transferrin, 66 nmol/liter insulin, 100 nmol/L dexamethasone, 1 nmol/L triiodothyronine, 3-isobutyl-1-methylxanthine (0.25 mmol/L), and rosiglitazone (10 µmol/L). The adipogenic medium was replaced after 3 days with maintenance medium (lacking 3-isobutyl-1-methylxanthine and rosiglitazone), which was changed three times per week. Every experiment was performed in fully differentiated adipocytes (namely 12 days of differentiation). The differentiation in adipogenic medium of stromal vascular fraction in mature adipocytes was verified by oil red O staining, a cytological stain used to detect neutral lipids, mainly triglycerides, and by peroxisome proliferator-activated receptor γ (PPARγ), leptin, and fatty acid binding protein-4 (FABP4) genes that are traditionally expressed in mature adipocytes, as previously described [35,36] (Figures S1 and S2).

### 4.2. RNA Extraction

Total RNA was extracted from human adipose tissue and adipocyte cultures on the 12th day of differentiation using RNeasy Lipid Tissue and RNeasy Mini Kit (Qiagen GmbH, Germany). Extracted RNA was quantified using NanoDrop technology (Fisher Scientific SAS, Illkirch Cedex, France) and immediately stored at −80 °C until needed. For DNAse treatment and reverse transcription, RNA (2 μg) was treated with DNase treatment and removal reagents (Ambion, Inc., Austin, TX, USA) using the manufacturer’s instructions. DNAse-treated RNA was reverse-transcribed for 1 h at 37 °C with 150 ng random hexamers, 0.5 mM dNTPs, 20 U of RNAsin Ribonuclease Inhibitor, and 200 U of M-MLV Reverse Transcriptase (Promega, Madison, WI, USA).

### 4.3. Detection of P2 Purinergic Receptor RNAs by Reverse-Transcriptase Polymerase Chain Reaction (RT-PCR)

PCR was performed at least on three independent biological replicates for each sample using specific primers and reaction conditions. The PCR was performed with HotStarTaq Master Mix Kit (Qiagen GmbH, Hilden, Germany) using 5 ng of cDNA for each amplification and including a positive reaction control (Table 1). Amplicon fragments were separated on a 2% agarose gel, stained with ethidium bromide and with a 100 bp ladder as a marker (Promega).

### 4.4. Sequencing

Specific amplifications of the P2 receptor mRNAs were confirmed by sequencing of the PCR fragments using ABI PRISM Big Dye Terminator Cycle Sequencing Ready Reaction Kits (Applied Biosystems, Forster City, CA, USA) using the following parameters of reaction: 95 °C 4 s, 50 °C 10 s, 60 °C 4 min for 25 cycles. Products were then purified using the Centri-sep spin columns (Applied Biosystems, Foster City, CA, USA) and tested by ABI 3100 Sequencing Analyzer (Applied Biosystems). The sequencing data were analyzed using Chromas Lite and sequences were identified using the Basic Local Alignment Search Tool program (www.ncbi.nlm.nih.gov, accessed on 21 September 2021).

### 4.5. Measurement of [Ca^2+^]^i^ in Human Adipocytes

Primary cultures of adipocytes were differentiated on glass coverslips and incubated with 2 µM fura-2/AM (Molecular Probes, Invitrogen) for 30 min at 37 °C in the dark, as previously described [33]. After incubation, cells were washed twice in the standard medium and suspended in 1 mL of a solution containing (in mM) 140 NaCl, 5.0 KCl, 1.0 MgCl_2_, 25 HEPES, and 5 glucose (pH 7.4) at 37 °C, with or without the addition of 1.0 mM CaCl_2_ (for standard or Ca^2+^-free medium, respectively). For [Ca^2+^]_i_, measurement coverslips were transferred to the recording chamber, maintained at 37 °C, and placed on the stage of an inverted epifluorescence microscope. Cells were stimulated with ATP or UTP in medium with or without calcium. Images of fura-2-loaded adipocytes with an excitation wavelength alternating between 340 and 380 nm were captured with a cooled CCD camera. After subtraction of background fluorescence, the ratio of fluorescence intensity at the two wavelengths was calculated. Ratio levels in groups of 10 to 15 individual cells per coverslip were analyzed using MetaFluor software package (Universal Imaging Corp., West Chester, PA, USA). All graphs are averaged responses from groups of 50–100 individual cells from representative single experiments. All experiments have been repeated on three separate occasions, and similar results were obtained. In parallel experiments fura-2-loaded adipocytes were incubated in Ca^2+^-free medium before stimulation. All experiments have been repeated on three separate occasions.

### 4.6. Measurement of IL-6 Concentration after Adipocyte Stimulation with ATP

The specific effects of ATP and other nucleotides on purinergic receptor activation in adipocytes have been tested using specific agonists. In the experiments examining the effects of extracellular ATP on IL-6 secretion, human adipocytes have been incubated in the presence of the appropriate stimulus and then the levels of IL-6 have been measured in conditioned medium by enzyme-linked immunosorbent assay (Human IL-6 Quantikine ELISA kit, R&D Systems). The assay sensitivity was less than 1.0 pg/mL, and the intra- and inter-assay coefficient of variations were 3.4% and 5.2%, respectively. In experiments evaluating the role of spontaneously released ATP from adipocytes in the stimulation of IL-6 secretion, apyrase from potato (Merck), an ATP-hydrolyzing enzyme, was added to the culture medium (3 units/mL).

### 4.7. Statistical Analyses

Statistical analysis was performed using GraphPad PRISM software (version 3.03; GraphPad Software Inc., San Diego CA, USA). The results are expressed as the mean ± SD. A paired *t*-test was applied to compare data from gene and protein expression obtained with the stimulation of different agonists. Data were analyzed with one-way analysis of variance followed by a post hoc analysis with Bonferroni adjustment of the *p* value. Values of *p* < 0.05 were considered to be statistically significant.

## 5. Conclusions

In conclusion, the present study demonstrates that human adipose tissue/adipocytes express different P2X and P2Y purinergic receptor subtypes. These receptors are functionally active, being responsive mainly to ATP but also to other purine and pyrimidine analogs. Furthermore, ATP seems to be constitutively released in the extracellular space where it stimulates adipocyte production of IL-6, a well-known pro-inflammatory cytokine inducing a low-grade inflammatory state.

The demonstration of the presence of multiple P2 purinergic receptor subtypes in adipose tissue that participate in regulating its functions could allow new possible targets for the discovery of new molecules to influence their activity and adipocyte functions, possibly also influencing the important clinical consequences of obesity.

## Figures and Tables

**Table 1:** List of primer sequences and experimental conditions in PCR.

Gene	Forward (5′-3′)	Reverse	Annealing Temperature (°C)	N. of Cycle	Expected Product (bp)	Positive Control
P2X1	TTCAGGTTTGCCAGGCACTTT	CCCCAAAGATGCCAATTCCA	59	40	160	PBMC
P2X2	GGTGTCATCGGGGTCATTAT	ACCTGAAGTTGTAGCCGTACGA	56	40	130	A-172
P2X3	CACCTATGAGACCACCAAGTCG	CGTCACGTAATCAGACACATCC	59	40	222	A-172
P2X4	TGTGATACCAGCTCAGGAGGAAAAC	GCATCATAAATGCACGACTTGAGGT	66	35	396	PBMC
P2X5	CTTCTCCAAAAGCAATGTGATGGAC	GATGAGTACCAGGTCGCAGAAGAAA	65	39	406	PBMC
P2X6	ATGGAATCCGCTTCGACATC	TCCTCCAGTAGAAATGGGCTTC	60	38	165	HEK-293
P2X7	TGATAAAAGTCTTCGGGATCCGTTT	CCTGGACAAATCTGTGAAGTCCATC	65	39	398	PBMC
P2Y_1_	ATGTGTGCTTTCAATGACAGGGTTT	TGTGGATGTGGGCATTTCTACTTCT	66	38	399	PBMC
P2Y_2_	GTGTGCATTCATGAGTGAGGAACC	ATCAGACACAGCCAGGTGGAACATA	68	40	313	PBMC
P2Y_4_	CCACCTGGCATTGTCAGACACC	GAGTGACCAGGCAGGCACGC	61	38	425	PBMC
P2Y_6_	CGCTTCCTCTTCTTGCCAACC	CCATCCTGGCGGCACAGGCGGC	62	36	365	Placenta
P2Y_11_	CTACAGAGCGTATAGCCTGGTGCTG	CCATGTAGAGTAGAGGGTGGACACA	70	39	365	PBMC
P2Y_12_	CATTCAAACCCTCCAGAATCAACAG	CGATCGATAGTTATCAGTCCCAGGAA	68	36	413	PBMC
P2Y_13_	AAAAACACTTTGGTGGCCGACTT	ACAGCACGATGCCCACATACAT	58	37	154	PBMC
P2Y_14_	CACTTCAAGACGACAAACG	GAATATCCATCCTGACACTCC	60	38	108	Placenta

## Data Availability

The data presented in this study are available on request from the corresponding author.

## Supplementary Materials

The following supporting information can be downloaded at: https://www.mdpi.com/article/10.3390/molecules27061913/s1, Figure S1: PPARγ, leptin and fatty acid binding protein-4 (FABP-4) mRNA expression in pre-adipocytes (white bars) and in fully differentiated adipocytes (after 12 days of differentiation in adipogenic medium, black bars) and Figure S2: Oil Red-O staining showing the differentiation of pre-adipocytes to mature adipocytes (40× magnification).

## Sample Availability

Samples of the compounds are not available from the authors.
