# Supplementary material for: Molecular and Pharmacological Evidence for the Expression of Multiple Functional P2 Purinergic Receptors in Human Adipocytes
Source: Molecules. 2022 Mar 16;27(6):1913. doi: 10.3390/molecules27061913 (PMC8954896; doi:10.3390/molecules27061913)
Supplement: Supplementary file 1 [file molecules-27-01913-s001.zip › molecules-1601368-supplementary.pdf]

**Supplementary materials.**

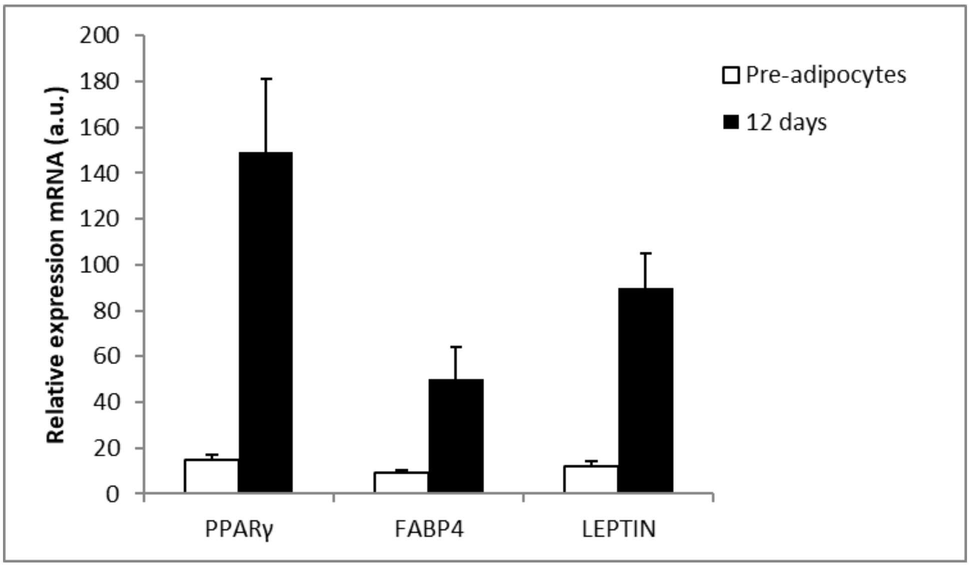


**Figure S1**. PPARγ, leptin and fatty acid binding protein-4 (FABP-4) mRNA expression in pre-adipocytes (white bars) and in fully differentiated adipocytes (after 12 days of differentiation in adipogenic medium, black bars).

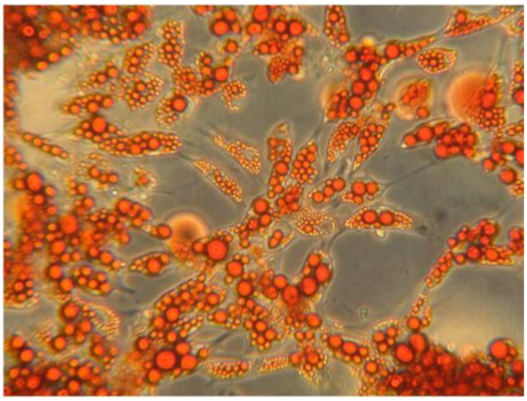

**Figure S2**. Oil Red-O staining showing the differentiation of pre-adipocytes to mature adipocytes (40x magnification).
